# Worldwide epidemiologic factors in pemphigus vulgaris and bullous pemphigoid

**DOI:** 10.3389/fimmu.2023.1159351

**Published:** 2023-04-25

**Authors:** Mattie Rosi-Schumacher, John Baker, James Waris, Kristina Seiffert-Sinha, Animesh A. Sinha

**Affiliations:** Department of Dermatology, University at Buffalo, Buffalo, NY, United States

**Keywords:** pemphigus vulgaris, bullous pemphigoid, prevalence, incidence, gender, age of onset, HLA-association

## Abstract

Autoimmune blistering diseases such as bullous pemphigoid (BP) and pemphigus vulgaris (PV) are complex, multifactorial, and polygenic diseases, whose exact pathogenesis is difficult to pinpoint. Research aimed at elucidating the associated epidemiologic risk factors of these two diseases has been hampered by their rare disease status. Further, a lack of centralization and standardization of available data makes the practical application of this information challenging. In order to collate and clarify the available literature we comprehensively reviewed 61 PV articles from 37 different countries and 35 BP articles from 16 different countries addressing a range of disease relevant clinical parameters including age of onset, sex, incidence, prevalence, and HLA allele association. The reported incidence of PV ranged from 0.098 to 5 patients per 100,000 people, while BP ranged from 0.21 to 7.63 patients per 100,000. Prevalence of PV ranged from 0.38 to 30 per 100,000 people and BP ranged from 1.46 to 47.99 per 100,000. The mean age of onset in patients ranged from 36.5 to 71 years for PV and 64 to 82.6 years for BP. Female-to-male ratios ranged from 0.46 to 4.4 in PV and 1.01 to 5.1 in BP. Our analysis provides support for the reported linkage disequilibrium of HLA DRB1*0402 (an allele previously shown to be associated with PV) and DQB1*0302 alleles in Europe, North America, and South America. Our data also highlight that HLA DQB1*0503 (also known to be associated with PV) appears in linkage disequilibrium with DRB1*1404 and DRB1*1401, mainly in Europe, the Middle East, and Asian countries. The HLA DRB1*0804 allele was only associated with PV in patients of Brazilian and Egyptian descent. Only two HLA alleles were reported as associated with BP more than twice in our review, DQB1*0301 and DQA1*0505. Collectively, our findings provide detailed insights into the variation of disease parameters relevant to PV and BP that can be expected to inform future work aimed at unraveling the complex pathogenesis of these conditions across the globe.

## 1 Introduction

Pemphigus vulgaris (PV) and bullous pemphigoid (BP) are rare autoimmune skin diseases associated with high morbidity and mortality. PV is characterized by flaccid blisters and erosions of the skin and mucous membranes due to the intra-epidermal separation of keratinocytes. Autoantibodies specific for desmosomal (desmoglein 1 and desmoglein 3) and potentially additional non-desmosomal (thyroid peroxidase, thyroglobulin, and more) moieties lead to a breakdown of intercellular adhesion structures responsible for epithelial integrity (1). On the other hand, BP is characterized by tense bullae of the skin and less oral involvement due to autoantibodies targeting hemi-desmosomal proteins (BP180 and BP230) anchoring the epidermis (2).

The etiopathogenesis of pemphigus and pemphigoid diseases is complex; in other words, it involves largely unknown environmental and polygenic genetic risk elements. One key genetic component that is required, but insufficient for disease development links to HLA class II molecules. In PV, the alleles DRB1*0402 and DQB1*0503 have been identified as PV-associated in the Ashkenazi Jewish and Caucasian population (3). While investigation into HLA associations in BP have been less extensive, DQB1*0301 has been highlighted in the literature multiple times as being associated with BP (4–7).

The accurate assessment of globally associated epidemiologic/genetic risk factors for pemphigus/pemphigoid diseases is hindered by the rare nature of these diseases, the complex nature of disease risk and development, the heterogeneity and individual variability of disease expression, as well as a lack of centralization and standardization of the available epidemiological, clinical, genetic data. A critical evaluation of the current status and fault lines in the informational landscape is required to formulate clear-eyed strategies aimed to advance our understanding of disease relevant etiopathogenic factors and ultimately improve patient access, care and outcomes.

The objective of the present study was to comprehensively evaluate and integrate information regarding the epidemiological features of patients with PV and BP across countries worldwide to provide enhanced insights regarding the presentation, demographics and genetic basis of disease. The parameters investigated included age of onset, sex, prevalence, incidence, and HLA associations.

As a note of context, Dr. Detlef Zillikens devoted his career to the investigation of autoimmune bullous disorders. His extensive track record of original research has served to provide many deep and original insights into the etiology, pathogenesis and management of these often devastating conditions. Dr. Zillikens has left an impressive legacy not only based on his individual accomplishments and collegiality, but perhaps most importantly due to his selfless commitment to his outstanding academic and clinical program at the University of Luebeck, world-wide collaborations, and his mentorship of countless junior faculty, fellows and students who will continue to carry the torch in our field. A number of the studies reviewed in this report were co-authored by Dr. Zillikens and members of his group (8–16). Several of the themes explored in this report are a direct extension of areas of inquiry initiated by Dr. Zillikens.

## 2 Methods

### 2.1 Pemphigus vulgaris

The data for the study was compiled by a thorough review of the literature. A total of 63 articles from 37 different countries published between 1974 to 2020 were mined from a PubMed online search for mention of search terms “Pemphigus and Incidence” and “Pemphigus and Prevalence” during July 2016 and August 2021 and “Pemphigus and HLA” during December 2016 and August 2021. Studies included were limited to English language only. Map visualization was achieved using the program www.mapchart.net.

Some studies were excluded from analysis due to the study populations having a mixture of PV and PF patients. We elected to exclude six studies that had populations less than 70% PV patients (17–22). While information on pemphigus foliaceous is often reported in conjunction with PV, we elected to focus on PV for this paper as the data on PF was often found in the context of “Pemphigus” as a whole, rather than separated on its own. Further, much of the information on solely PF was in the context of fogo selvagem, or endemic PF, which has unique features compared to PF. While a subject of interest, unfortunately most studies used in this manuscript were not derived from national registries, but rather from independent institutions or regional groups of institutions. National registries may, in the future, serve as an advantageous tool for collecting information on these rare diseases.

### 2.2 Bullous pemphigoid

Data for this study were obtained *via* a comprehensive review of the literature. A total of 35 articles from 16 different countries between 1992 and 2021 were obtained from an online search of PubMed. Terms searched included “Bullous Pemphigoid and Incidence”, “Bullous Pemphigoid and Prevalence”, “Bullous Pemphigoid and Epidemiology”, and “Bullous Pemphigoid and HLA” between September 2021 and January 2022. Studies were limited to those written in the English language. Maps were created using the website www.mapchart.net.

## 3 Results and discussion

### 3.1 Prevalence

**Figure 1:**
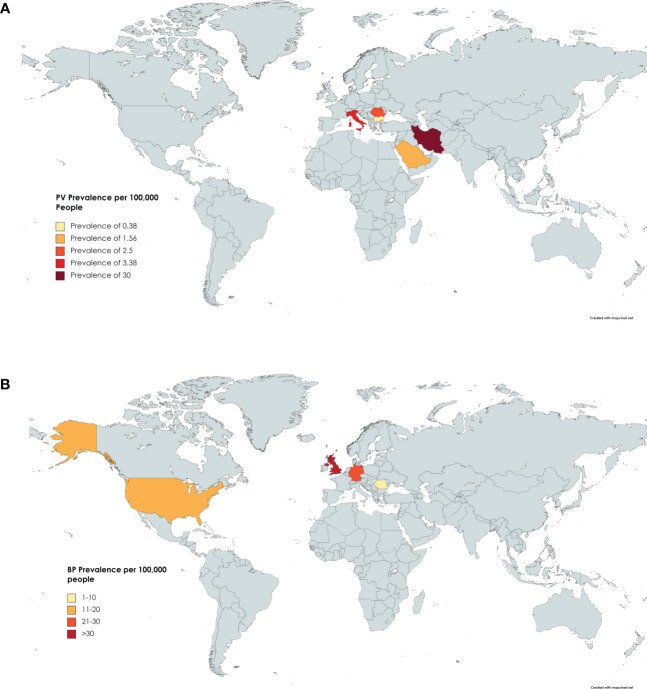
Prevalence by Country. **(A)** Countries include Iran, Italy, Germany, Romania, Saudi Arabia, and Bulgaria. Darker shading signifies higher PV prevalence. Specific values can be found in **Table 1A**. **(B)** Countries reporting BP prevalence include the USA, UK, Romania, and Germany. Specific Values can be found in **Table 1B**.

Six studies reported on the prevalence of PV in Iran (23), Italy (24), Romania (8), Saudi Arabia (25), Germany (12), and Bulgaria (26). The highest prevalence was found in Iran (23) with 30 per 100,000 people per year, while the lowest prevalence was found in Bulgaria (26) with 0.38 per 100,000 people per year (**Figure 1A** and **Table 1A**).

**Table 1A:** Prevalence of PV by Country.

Country	Prevalence of PV per 100,000 per year	Citation
Bulgaria	0.38	(26)
Saudi Arabia	1.56	(25)
Romania	2.5	(8)
Italy	3.38	(24)
Germany	9.48	(12)
Iran	30	(23)

Four studies reported on the prevalence of BP in Romania (8), Germany (12), the UK (27), and the USA (28). The highest prevalence was seen in the UK with 47.99 cases per 100,000 people per year (27). The lowest prevalence was seen in Romania at 1.46 cases per 100,000 people per year (8). The mean prevalence was 21.84 per 100,000 people, but the relatively small number of studies included in this analysis may serve to obnubilate the significance of this mean (**Figure 1B** and **Table 1B**).

**Table 1B:** Prevalence of BP by Country.

Country	Prevalence of BP per 100,000 per year	Citation
Romania	1.46	(8)
USA	12	(28)
Germany	25.9	(12)
UK	47.99	(27)

Figures on disease prevalence were the least available form of epidemiological data reported in the literature, likely due to the fact that prevalence data can be difficult to estimate in large populations in the case of rare diseases. Two factors that likely contribute to the variations in reported disease prevalence include regional discrepancies in (the largely unknown) disease predisposing genetic and/or environmental elements between different ethnic groups, as well as additional disease modifying factors, such as access to specialized health care facilities and expert care, that impact disease reporting practices in differing countries. Creating broad disease registries, coordinated to encompass standard reporting criteria implemented internationally could prove valuable in acquiring more accurate prevalence values.

### 3.2 Incidence

**Figure 2:**
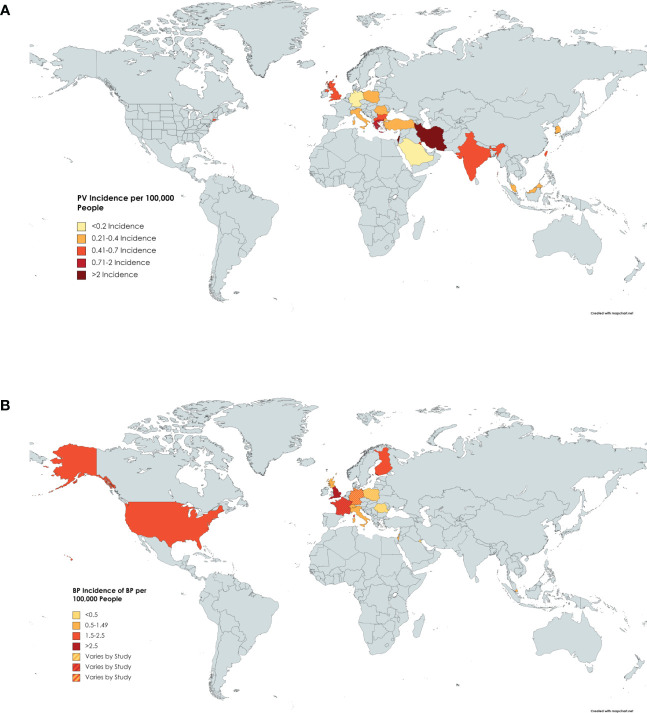
Incidence of PV by Country. **(A)** Darker shading of countries signifies higher incidence of pemphigus vulgaris. Specific incidence values can be found in **Table 2A**. **(B)** Darker shading of countries signifies higher incidence of BP. Specific incidence values can be found in **Table 2B**.

Twenty-one papers from 1974 to 2018 reported on the incidence of pemphigus vulgaris (8, 11, 23–26, 29–44). The overall incidence of PV varied significantly in various ethnic populations. The highest incidence was found in Iran with 5 per 100,000 people per year (31). The lowest incidence was found in Germany with 0.098 per 100,000 people per year (11). The highest incidences seem to cluster around the Middle East, with higher incidence trending south on the map towards the Tropic of Cancer (**Figure 2A** and **Table 2A**).

**Table 2A:** Incidence of PV by Country.

Country	Incidence of PV per 100,000 per year	Citation
Germany, Lower Franconia and Mannheim	0.098	(11)
Macedonia, Ethnic Albanians	0.1	(43)
Saudi Arabia	0.16	(25)
Malaysia	0.2	(29)
South Korea	0.21	(37)
Poland	0.24	(44)
Turkey	0.24	(42)
Italy	0.25	(24)
Romania	0.4	(8)
Hartford, Connecticut, US, overall adults	0.42	(41)
India (Thrissur District)	0.44	(34)
Macedonia	0.44	(43)
South India	0.44	(34)
Kuwait	0.46	(38)
Bulgaria	0.47	(26)
Taiwan	0.47	(33)
Macedonia, Ethnic Macedonians	0.51	(43)
Jerusalem, Non-Ashkenazi	0.61	(39)
Iran	0.67	(40)
Guadeloupe	0.696	(32)
UK	0.7	(36)
Macedonia during unrest	0.89	(43)
Greece	0.93	(35)
Iran	1	(23)
Jerusalem Jews	1.61	(39)
Macedonia, Roma (Gypsies)	2.4	(43)
Jerusalem, Ashkenazi	2.7	(39)
Hartford,Connecticut, US, Jewish Adults	3.2	(41)
Iran	5	(31)

Seventeen papers published between 1995 and 2022 reported on the incidence of BP (8, 9, 16, 20, 27, 36, 38, 45–55) which was highest in the UK with one study reporting an incidence of 7.63 cases per 100,000 people per year (27) (**Figure 2B** and **Table 2B**). Kuwait had the lowest incidence at 0.21 cases per 100,000 people per year (45). The mean incidence worldwide was 2 cases per 100,000 people per year.

**Table 2B:** Incidence of BP by Country.

Country	Incidence of BP per 100,000 per year	Citation
Kuwait	0.21	(38)
Romania	0.25	(8)
Kuwait	0.26	(45)
Poland	0.447	(46)
Poland	0.738	(47)
Singapore	0.76	(48)
Italy	1	(49)
Israel	1.14	(50)
Switzerland	1.21	(20)
Germany	1.34	(9)
Scotland	1.4	(51)
Finland	1.7	(52)
Germany	1.96	(16)
France	2.17	(53)
Olmstead County, Minnesota, US	2.4	(54)
UK	4.3	(36)
France	7	(55)
UK	7.63	(27)

While there is more data available for incidence than prevalence in both of these diseases, there still exists both a paucity and notable variance in the worldwide data. Although there are reports of increasing rates of autoimmune diseases worldwide, there are currently no reports endorsing this phenomenon in PV (56). BP, however, has displayed an increase in incidence over the past 2 decades. Explanations for this include increasing diagnostic capability and increased life expectancy (57).

Both PV and BP are known to be multifactorial in origin and the wide fluctuation among data worldwide supports the hypothesis that geographic location, and associated racial and ethnic population differences linked to genetic risk factors can influence the incidence of these diseases. Other confounding variances in socioeconomic status, access to healthcare, and the role of specific environmental and “exposome” factors are no doubt impactful as well, but cannot easily be accounted for. Nevertheless, broad trends in the data connote that careful analysis in future work focused on isolating the role of specific genetic and environmental risk elements within specific ethnic groups and geographic locations will be essential in lifting the veil on the fuller scope of meaningful agents that undergird and shape the spectrum of disease.

### 3.3 Gender

**Figure 3:**
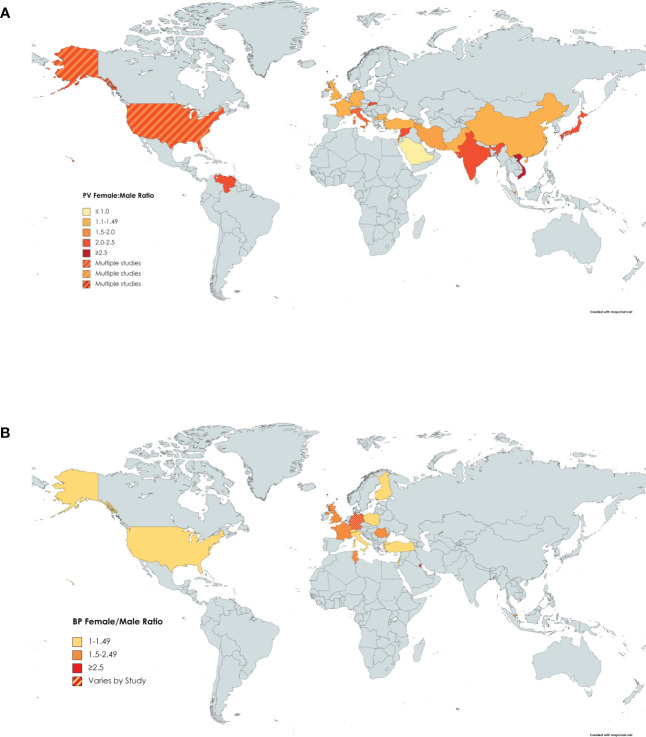
Gender Ratio of PV by Country. Darker shading signifies higher female:male gender ratio of **(A)** PV or **(B)** BP. Countries with more than one shaded color signify that more than one study had results that fell into different categories.

Twenty-five papers were reviewed that reported on the gender ratio of females to males in PV (10, 23–26, 31, 34, 36, 40, 42, 43, 58–72). The lowest ratio, less than one, indicating there were more males affected than females, was found in Saudi Arabia with a ratio of 0.46 (25). All other ratios were greater than one, indicating most studies found that more females were affected than males. The greatest ratios were found in Mexico with a ratio of 4.4 (72). The mean of all the ratios from the studies was 1.74 (**Figure 3A**).

For BP, we reviewed twenty papers reporting on the gender ratio (8, 9, 14, 18, 20, 36, 38, 42, 45, 47–54, 73–75). Finland had the lowest female to male gender ratio of 1.01 (52), while two separate studies in Kuwait reported the highest gender ratios of 5.1 (45) and 5.75 (38). The mean female to male ratio worldwide was 1.87 (**Figure 3B**).

As noted, Kuwait displayed by far the highest female to male ratio in BP. In two separate studies, the female to male ratio was recorded as being over 5 (38, 45). The next highest BP female to male gender ratio was 2.5, found in Germany by Ständer et al. (14) in a small subgroup of patients with localized BP. The extent to which this elevated gender ratio in Kuwait is a reflection of true regionally linked differences in reported ratios from other worldwide locations remains to be determined. Unfortunately, there are no studies from surrounding countries in proximity to Kuwait that could be used for comparison. However, the fact that two separate studies from two years apart reported similarly high female to male ratios in Kuwait lends credence to their validity, rather than being a statistical anomaly. Additional studies on gender ratio from countries in the Middle East would help to corroborate this data, whose potential disease significance awaits further exploration.

Overall, both PV and BP, like the majority of autoimmune diseases, show a consistent bias towards females. There are multiple theories that posit explanations for this bias including effects from the menstrual cycle, hormonal effects on the microbiome, and immune-related genes located on the X-chromosome (76). However, the specific mechanisms of female skewing in autoimmune blistering diseases, and autoimmunity in general remain enigmatic. It is also unclear the extent to which female patients differ from males in terms of key disease relevant factors such as genetic predisposition, immune dysregulation including autoantibody levels, and clinical course. However, within the context of PV specifically, Naseer et al. found that female patients had a later age of onset as well as higher rates of mucosal disease than male patients (77). Further investigation *via* retrospective as well as longitudinal prospective analyses are needed to better define gender specific differences regarding autoimmune risk and expression.

### 3.4 Age of onset

**Figure 4:**
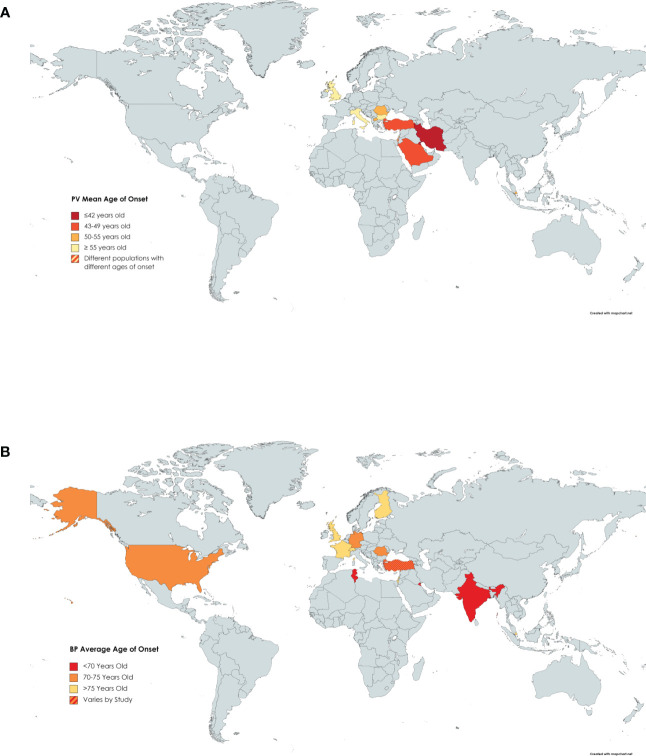
Age of Onset of PV by Country. Darker shading signifies a younger age of onset of **(A)** PV or **(B)** BP. Countries with more than one shaded color signify that more than one study had results that fell into different categories.

Fourteen papers were reviewed that presented information on the mean age of onset in PV patients enrolled in their study (8, 23–26, 31, 36, 38, 40, 42, 43, 58, 64, 71). The ages ranged from 36.5 years in Kuwait (38) to 71 years in the United Kingdom (36), with the average age across these studies being 49.8 years (**Figure 4A**). The average age of onset of PV was higher than several autoimmune diseases such as SLE, systemic sclerosis, and psoriasis (78). PV shared a similar age of onset to autoimmune diseases such as rheumatoid arthritis and Sjogren’s syndrome which tend to occur in the mid to late 40’s (78).

We reviewed 16 papers that reported the mean age of onset of BP patients enrolled in investigative studies (8, 9, 18, 20, 36, 38, 42, 45, 48, 50–54, 79, 80). Ages of onset ranged from 64 (42) to 82.6 (53) years with an average of 73.4 years (**Figure 4B**). This later age of onset in BP relative to PV is consistent with the fact that BP is known as a disease of the elderly.

Interestingly, both PV and BP both tend to have lower average ages of onset in southwestern Asia. PV was also found to generally have a higher incidence and prevalence in this region, pointing towards the possibility that there are distinct genetic risk factors specific to populations in that region. On the other hand, there is minimal data on BP incidence and prevalence in this region of southwest Asia.

The genetic, environmental, and ultimately the immunologic factors that arbitrate the generally late onset of the majority of autoimmune conditions, and specifically the predominant age of onset window in PV and especially BP are nebulous. Potential explanations include the erosion of immune tolerance over time, and an accumulating environmental burden that evolve a patient’s “umwelt” in later years.

### 3.5 HLA association

**Figure 5:**
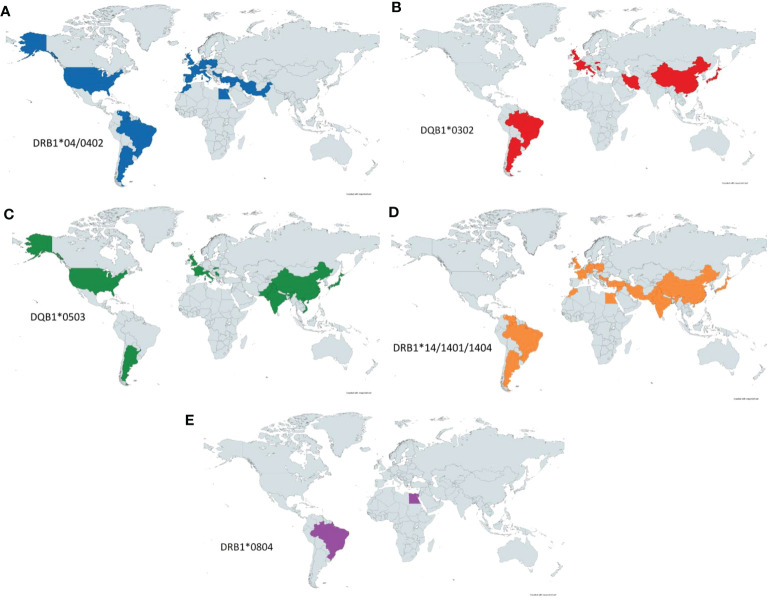
PV-HLA Associations Reported by Country. Known HLA associated alleles as reported by country. Significant overlap is seen between DRB1*0402 **(A)** and DQB1*0302 **(B)** as these alleles have previously been shown to be in linkage disequilibrium. DQB1*0503 **(C)** is seen throughout the world but is notably concentrated in eastern and southern Asia. DRB1*14 **(D)** is seen diffusely throughout the eastern hemisphere as well as South America. Lastly, DRB1*0804 **(E)** has been more rarely reported in PV but has been noted in Egypt and Brazil.

Analysis of the aggregated HLA data on PV revealed several trends, one of which highlights the evident linkage disequilibrium between HLA DRB1*0402 (10, 59–61, 63, 66, 68, 81–90) and DQB1*0302 alleles (60, 83, 86, 89, 91–94). HLA DRB1*0402 shows clustering in the Middle East (10, 59, 61, 68, 88, 89) and around Europe (10, 60, 85–87, 90, 95), with migratory spread into North (66) and South America (81, 82, 84) (**Figure 5A**). This genetic undercurrent is consistent with historic Ashkenazi Jewish and Mediterranean population migration to North America. DQB1*0302 allele distribution echoes similarly worldwide, promulgating support for the actuality of linkage disequilibrium between the DRB1*0402 and DQB1*0302 alleles (69) (**Figure 5B**). DQB1*0302 alleles are also prevalent in Asian countries such as China and Japan.

HLA DQB1*0503 (60, 67, 71, 84, 86, 92, 93, 95–98) shows clustering in western Europe (60, 86, 93, 95), North (67) and South America (84) as well as South and East Asia (India, Pakistan, China, Japan) (71, 96–99) (**Figure 5C**). A similar distribution is adhered to by DRB1*1404 and DRB1*1401 (10, 59, 60, 65, 68, 81, 82, 84, 89, 90, 93, 95–97, 100, 101) as seen in **Figure 5D**, again likely as a reflection of linkage disequilibrium between the DQB1*0503 and DRB1*14 alleles. DRB1*14 alleles are also found in the Middle East (10, 59, 68, 89, 96) and Brazil (81).

The HLA allele DRB1*0804 is predominantly detected in the Brazilian (102) and Egyptian (10) populations (**Figure 5E****)**. Both DRB1*0804 and DRB1*14 have been reported among PV patients in Brazil and Egypt, although further analyses will be required to certify the strength of these proclaimed diseases linkages.

**Figure 6:**
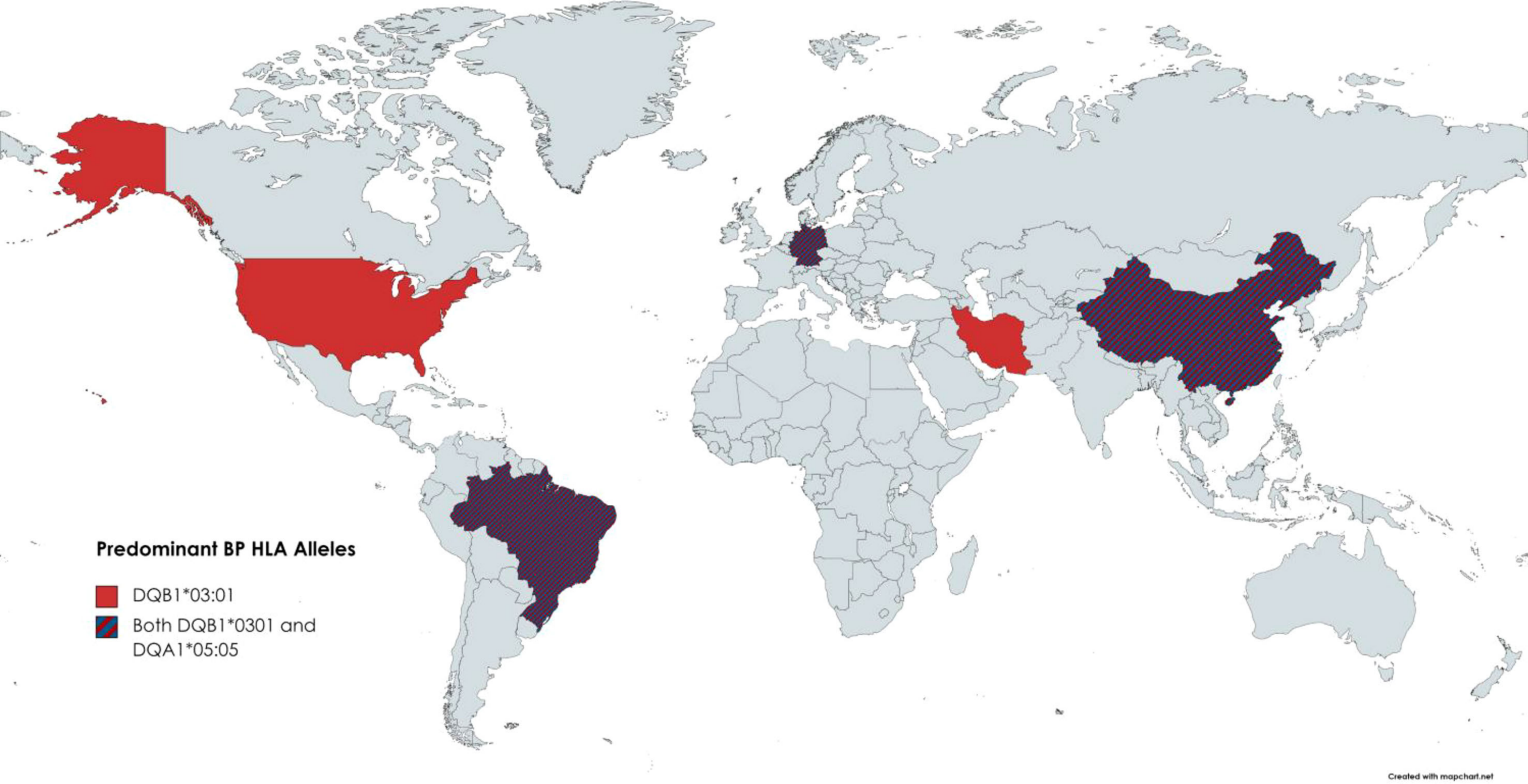
BP-HLA Associations reported by Country. Known HLA associations reported by country. The only two alleles repeated more than twice were DQB1*0301 and DQA1*0505.

Fewer studies were available on HLA and BP; we reviewed 9 papers that reported on HLA predominance among BP patients (**Figure 6**) (4–6, 13, 103–107). Of the nine articles that include information on HLA alleles of BP patients, only 2 HLA alleles were repeated more than twice across studies: DQB1*0301 and DQA1*0505.

DQB1*0301 came up as being associated with BP as early as 1996 and was reported in five countries. DQB1*03:01 has been included in several publications as an implicated factor in drug-induced BP. Antigen presentation by the DQB1*03:01 molecule has also been shown to boost T-cell avidity to several epitopes of BP180, a major autoantigen associated with bullous pemphigoid (108). Regarding the significance of DQA1*05:05 being overrepresented in BP patients, there is a need for follow-up inquest.

HLA genetics, global allele distribution and migrations, ethnic and disease associations are immensely complex. Yet the importance of HLA in determining disease risk, the specificity of the autoimmune attack and the final clinical presentation of disease cannot be overstated. A clearer discernment of true HLA-disease associations in autoimmune blistering diseases would benefit from robust, multinational databases that accelerate the collection of epidemiologic and genetic information at a larger scale in order to sufficiently power detailed multivariate analyses and improve the calculation of disease risk. Ultimately, studying the physical structure of each of the key HLA molecules associated with these diseases will be required to understand how a patient’s genetic profile translates to immune (dys)functionality to induce aberrant immune responses resulting in skin pathology.

## 4 Conclusions

**Figure 7:**
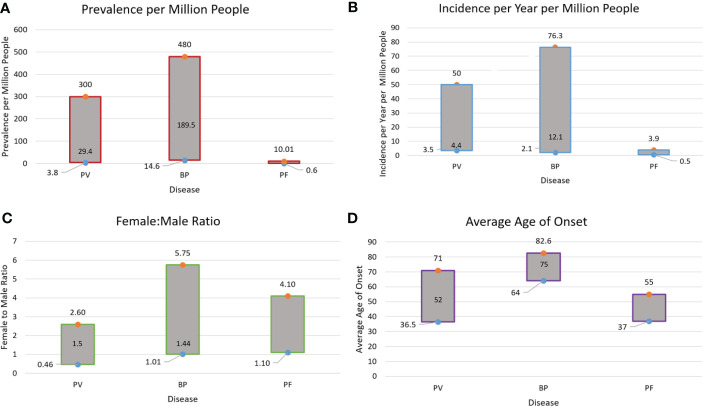
Summary of Prevalence **(A)**, Incidence **(B)**, Gender Ratio **(C)**, and Age of Onset **(D)** Ranges for PV, BP and Pemphigus Foliaceous (PF). Data on PF incidence and prevalence are sparse in the literature. This is due to the fact that PF is seldomly studied independently, while studies that report on Pemphigus as a singular disease category (including PV and PF) do not provide data individually on disease subtypes. The median values for age of onset, gender ratio, prevalence, and incidence in PV and BP are noted within the bars.

At present, available epidemiologic information on PV and BP is sparse worldwide. Existing data on the overall ranges of prevalence, incidence, gender ratio, and age of onset for PV, BP, and pemphigus foliaceous (PF) are summarized in **Figure 7** (8, 11, 12, 15, 17, 23, 25–27, 31, 36, 38, 42, 52, 53, 71, 109, 110). PF data were added as this condition closely relates to PV in terms of etiology and clinical expression, and the two are often reported together in studies. Yet, there remain gaps regarding epidemiology, and even HLA associations for both pemphigus and pemphigoid disease.

Moving forward, it will be important to develop concerted and coordinated efforts to compile more complete, comprehensive global datasets to hurdle current barriers impeding the study of ethnicity, genetic background, geographic location and environmental elements that impact disease risk and expression. While envisioning and effecting such an endeavor is undoubtedly challenging, we are at the precipice of emerging technology conceived to leverage digital health care information gathering and communication platforms, including mobile applications, with the potential to escalate clinical investigation to previously unattainable levels and surmount the difficulties of unpacking the complexities of multifactorial diseases. On this basis, we can expect to further uncover the mechanisms of disease risk and clinical heterogeneity, and instruct the formulation of more informed genetic and immunologic research that upraises the development of superior strategies to avert and remedy the autoimmune condition.

## Author contributions

AS and KS-S devised the project and guided data collection. MR-S, JW, and JB performed data selection and analysis. All authors contributed to writing and critical revision of the manuscript. All authors contributed to the article and approved the submitted version.
